# An Unusual Course of Segmental Renal Artery Displays a Rare Case of Hilar Nutcracker Phenomenon

**DOI:** 10.1155/2015/249015

**Published:** 2015-09-13

**Authors:** Devendra A. Sawant, Thomas F. Moore

**Affiliations:** ^1^School of Anatomical Science, Alderson Broaddus University College of Medical Science, Philippi, WV 26416, USA; ^2^School of Physician Assistant Studies, Alderson Broaddus University College of Medical Science, Philippi, WV 26416, USA

## Abstract

Nutcracker phenomenon or renal vein entrapment is classically seen as a compression of renal vein in between abdominal aorta and superior mesenteric artery with patients being asymptomatic or clinically manifested in the form of nutcracker syndrome as proteinuria, hematuria, flank pain, pelvic congestion in women, and varicocele in men. In this report, we are presenting a case of rare variant of nutcracker phenomenon along with brief review of anatomy, pathophysiology, public health, and clinical significance of nutcracker syndrome. On a routine dissection of an adult male cadaver, we noticed an unusual arrangement of the structures at the hilum of the left kidney showing entrapment of renal vein between left anterior inferior and posterior segmental renal arteries. The variation in the course of left anterior inferior segmental renal artery leads to compression of left renal vein at renal hilum. Therefore, we have named this rare abnormal anatomical entity as hilar nutcracker phenomenon. The structures in the right renal hilum are normal. The objective of this paper is to report an unusual but important variant of nutcracker phenomenon and also give collective knowledge of such anatomical variations in renal vasculature that will help in diagnosing and treating such rare renal disorder.

## 1. Introduction

The renal vein entrapment, also referred as nutcracker phenomenon is most commonly seen as a compression of renal vein in between abdominal aorta and superior mesenteric artery and the resulting clinical scenario is known as nutcracker syndrome. The nutcracker phenomenon could be asymptomatic or could give rise to nutcracker syndrome with clinical manifestations such as proteinuria, hematuria, pain, or discomfort in flank/abdominal/lumbar region, congestion in pelvic region in female, and varicocele in male patients. This usual form of nutcracker phenomenon or syndrome has also been termed as anterior or classic nutcracker phenomenon or syndrome [1–4]. The first description of this pathological disorder was given by the anatomist Grant in 1937, whereas the first presentation as a clinical case was reported by El Sadr and Mina in 1950 and then by Chait et al. in 1971, who described this anatomical pattern of the aorta and the superior mesenteric artery as two arms or handles of a “nutcracker” compressing renal vein in between [3, 5–8]. However, it was de Schepper who termed this phenomenon the “nutcracker syndrome” in 1973 [1, 3, 9].

Apart from the classic anterior nutcracker syndrome, there are other rare variants such as posterior or retroaortic nutcracker syndrome and combined nutcracker syndrome. The retroaortic or posterior nutcracker syndrome refers to the left renal vein compression in between the abdominal aorta and vertebral spine. The combination of anterior and posterior nutcracker syndrome with duplication of the left renal vein and compressing either the anterior branch between the abdominal aorta and superior mesenteric artery or posterior branch between the abdominal aorta and vertebral spine has been be presented as combined nutcracker syndrome [2]. Also, Basile et al. have presented a clinical case of left renal vein compression arising due to aberrant renal artery close to inferior vena cava leading to stenosis and renal hypertension [2]. Polguj et al. have also reported a lateral or anterolateral nutcracker phenomenon describing a left renal vein compression as it moves between the superior mesenteric artery and the right renal artery [8].

The symptoms of nutcracker syndrome can also occur due to renal vein undergoing compression from fibrolymphatic tissue, retroperitoneal tumor, para-aortic lymphadenopathy, pancreatic tumor, abdominal aortic aneurysm, and anatomical variation of testicular artery such as overarching leading to entrapment of renal vein. The nutcracker syndrome should not be confused with the Wilkie's syndrome or superior mesenteric artery syndrome, which is referred as duodenum getting entrapped and compressed in between abdominal aorta and superior mesenteric artery [3]. The clinical diagnosis of the nutcracker syndrome can be done by doppler ultrasound using contrast, computed tomography or magnetic resonance angiography. However, the gold standard in the diagnostic investigation is evaluating renocaval pressure gradient by retrograde venography or phlebography. The assessment of pressure gradient in between renal veins can also be used to confirm the diagnosis of obstruction in renal vein and assess the severity of the obstruction as well as monitor therapeutic progress while clinically managing patients with nutcracker syndrome [2, 4, 10–12].

The patients diagnosed with nutcracker syndrome should be managed according to their age and clinical symptoms of the patient. Usually young patients with less than 18 years of age are more frequently treated conservatively, since majority of such patients will show complete remission in their clinical symptoms including hematuria. Nutcracker syndrome patients with mild hematuria or with spontaneous resolution of hematuria can be managed with conservative therapy. However, surgical intervention for correcting anatomical anomaly including procedure such as renal autotransplantation is indicated for patients with significant pain and severe, persistent life threatening hematuria [1, 8, 13, 14].

## 2. Public Health/Epidemiological Data

The nutcracker syndrome is not a hereditary disorder, but reports have been presented showing sporadic cases in the siblings [3, 15]. The incidence of anterior or classic nutcracker syndrome is higher than the retroaortic nutcracker syndrome. The incidence of retroaortic nutcracker syndrome varies from 0.8% to 7.1% [4, 16]. The nutcracker syndrome, once considered to be a rare entity, has recently been shown to be diagnosed increasingly among patients with hematuria or proteinuria. However, the exact prevalence rate of nutcracker syndrome cannot be formulated due to variability in symptoms and lack of standardized criteria for diagnosing nutcracker syndrome [3, 4]. There is also insufficient epidemiological data showing prevalence and incidence rates among various demographic groups. Similarly, lack of advanced imaging techniques and diagnostic modalities in the less developed part of the world would make such disorders difficult to diagnose. And therefore, due to nonreporting of this disease condition, the prevalence and incidence rates of nutcracker syndrome are still very low in these populations [13].

The nutcracker syndrome can be seen among all the age groups from pediatric to geriatric patients with increased prevalence rate among young adults [4, 14]. Some reports have stated that rapid growth spurt with increase in body height and the maturation of the vertebral bodies during puberty could lead to decrease in angle formed by the superior mesenteric artery and abdominal aorta resulting in entrapment and compression of renal vein [4, 14]. According to some studies, there could be gender dimorphism in nutcracker syndrome, with slightly more predisposition towards female gender especially affecting middle aged women. However, one study showed that no such difference exists in prevalence rate between males and females [4, 14, 17]. Also, individuals with low body mass index along with lordosis and decreased fat in retroperitoneal and mesenteric region have been shown to increase the risk factor in compressing renal vein leading to nutcracker syndrome [4, 18]. Similarly, reports have also suggested that, in pregnancy, compression of the large veins by gravid uterus could be a contributing factor in the pathogenesis of the right-sided nutcracker syndrome [3, 4, 19].

## 3. Clinical Significance

The nutcracker phenomenon could be asymptomatic or could give rise to nutcracker syndrome with clinical symptoms such as proteinuria, hematuria, abdominal or flank or lumbar discomfort or pain, varicocele in the men, and pelvic congestion in the women that include vulvar and pelvic varices along with dyspareunia, dysuria, and dysmenorrhea [8, 20]. The hematuria that includes both gross and microscopic hematuria followed by pain in abdominal and flank regions is the most common symptom reported in the patients with nutcracker syndrome. Similarly, about 5.5% to 9.5% of men with nutcracker syndrome get affected by varicocele with predominance on left side [3, 21].

The nutcracker syndrome due to left renal vein compression results in medial or mesoaortic narrowing and lateral or hilar dilatation [8]. The increased pressure in the left renal vein and the renal hilum dilatation lead to congestion of the left kidney [8]. The compression of renal vein can also manifest as gonadal vein, renal pelvis, and ureter varicosities leading to flank or abdominal discomfort or pain, albuminuria, hematuria, abnormal menstruation in the women, and varicocele in the men. Less frequently, renal vein compression can also present with symptoms involving gastrointestinal system, chronic fatigue syndrome in children and adolescents, and orthostatic proteinuria [8]. The gonadal vein or ovarian vein pain syndrome, a form of pelvic congestion syndrome, can manifest as flank or abdominal pain that sometimes can also involve posteromedial part of the thigh and buttock region. The pain can also show exacerbation while sitting, standing, walking, or doing movement such as riding in a moving vehicle. The flank pain can also occur from ureteral colic due to passage of blood clots through the ureter [3]. Routinely, patients with nutcracker syndrome can also present with varicose veins in their lower extremities due to renal vein compression causing renal to gonadal vein reflux [1, 20]. The compression of renal vein leads to increase in pressure within the renal circulation which in turn leads to formation of plexus of abnormal hypertensive venous collaterals at the renal pelvis causing gross or microscopic hematuria [8]. However, such gross or microscopic hematuria in nutcracker syndrome seldom causes anemia in patients [3].

Apart from its anatomical classification, the nutcracker syndrome can be clinically differentiated into two subtypes, such as typical with renal manifestation and atypical with urologic manifestation. The typical clinical nutcracker syndrome with renal manifestation includes gross or microscopic hematuria and orthostatic proteinuria with or without flank pain. And the atypical clinical nutcracker syndrome with urologic manifestation includes fatigue, flank/abdominal pain, orthostatic intolerance, dysmenorrhea and dyspareunia in women, and varicocele in men [21]. It is also proposed that nutcracker syndrome can be differentiated clinically into three subtypes, such as idiopathic hematuria, orthostatic proteinuria showing protein levels in urine more than 400 mg/dL, and orthostatic intolerance that leads to serious morbidity and interference with daily activities causing disability in living normal life [3].

Nutcracker syndrome patients with orthostatic disturbance can also manifest systemic clinical symptoms such as headache, abdominal pain, fainting, and tachycardia [1]. Although systemic hypertension is not considered in the clinical manifestation of the nutcracker syndrome, some reports have described the presence of renin dependent hypertension in the patients with nutcracker syndrome [22]. Similarly, nutcracker syndrome is also known to be associated with various other clinical disorders such as IgA nephropathy, membranous nephropathy, idiopathic hypercalciuria, Henoch-Schonlein purpura, and familiar Mediterranean fever [3, 22]. There are clinical reports of patients presenting with nutcracker syndrome combined with other syndromes such as median arcuate ligament syndrome and May-Thurner syndrome [3]. Thus, the knowledge of anatomical variations in renal vasculature such as nutcracker syndrome will be very useful in treating patients with rare renal disorders. It will also serve as a valuable tool for surgeons performing and managing complicated renal surgeries. In this paper, we are reporting a rare case of nutcracker phenomenon that showed left renal vein compression by getting entrapped between anterior inferior and posterior renal segmental arteries at the hilum of the left kidney.

## 4. Case Report

**Figure 1:**
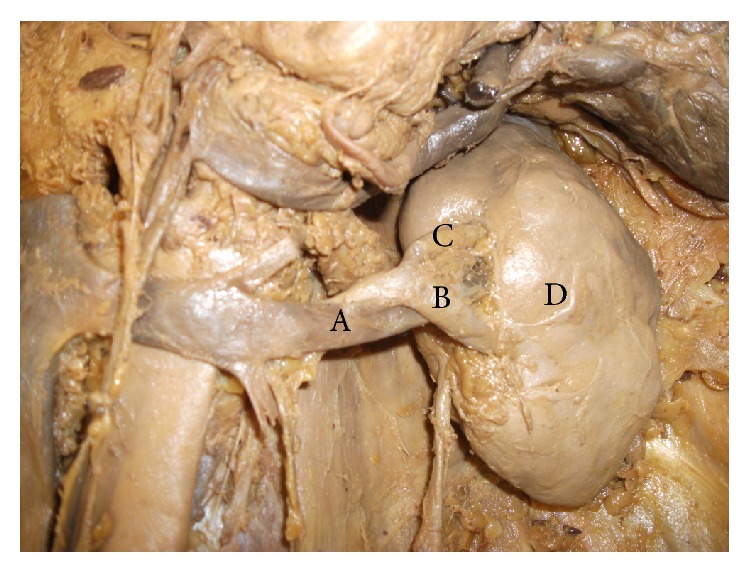
Left renal hilum (anterior view). (A) Left renal vein, (B) left anterior inferior segmental renal artery, (C) left anterior superior segmental renal artery, and (D) left kidney.

**Figure 2:**
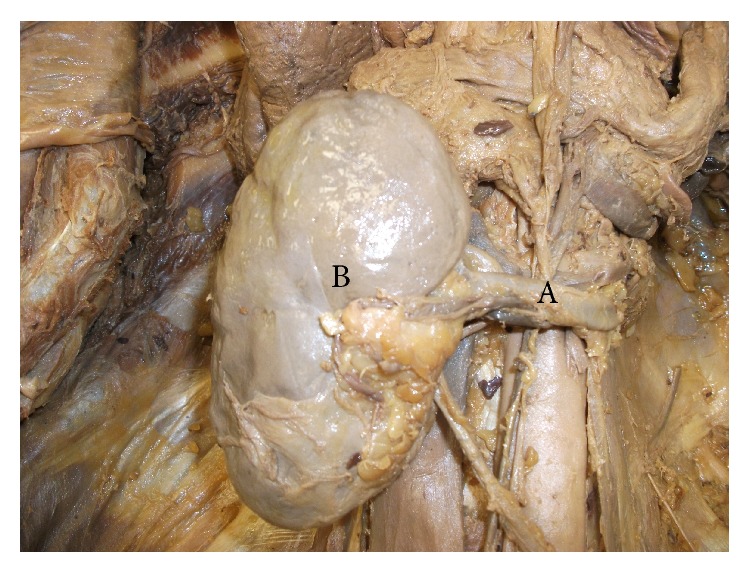
Left renal hilum (posterior view). (A) Left posterior segmental renal artery, (B) left kidney.

On a routine dissection of an adult male cadaver, we noticed a variant of nutcracker phenomenon due to an unusual arrangement of the structures at the hilar region of the left kidney leading to left renal vein compression by overlapping anterior inferior segmental branch of a left renal artery. The normal pattern of structures passing through the hilum from anterior to posterior is renal vein, and then renal artery followed by the pelvis of the ureter [23]. According to Terminologia Anatomica, the renal artery divides into five segmental arteries such as superior, anterior superior, anterior inferior, inferior, and posterior segmental arteries near the hilum to supply respective renal segments [24]. However, there can be considerable amount of variation from this usual pattern of arterial supply to the renal segments [23]. In this reported cadaveric body, the left main renal artery after arising from the aorta runs posterior to the renal vein. In the prehilar region around 3 to 4 cm away from the hilum of left kidney, the left main renal artery divides into two segmental arteries, namely, anterior and posterior segmental arteries. The anterior segmental artery during its course gives an anterior superior segmental artery and then continues further as anterior inferior segmental artery. The anterior segmental renal artery, after arising from the main left renal artery posterior to left renal vein, overrides the left renal vein in a serpentine fashion to enter the hilum of the left kidney anterior to left renal vein as anterior inferior segmental artery. During this anatomical course of the left anterior inferior segmental renal artery, the left renal vein gets entrapped between two anterior inferior and posterior segmental renal arteries. This unusual serpentine course of the left anterior inferior segmental artery tends to compress the left renal vein demonstrating a nutcracker phenomenon at the hilum of the left kidney on its anterior view (Figure 1). And from the posterior view, the posterior segmental artery can be seen entering the hilum of left kidney (Figure 2). So, in this reported anatomical abnormality, the two arms of the “nutcracker” are represented by the left anterior inferior and posterior segmental renal arteries with renal vein getting entrapped and compressed in between.

**Figure 3:**
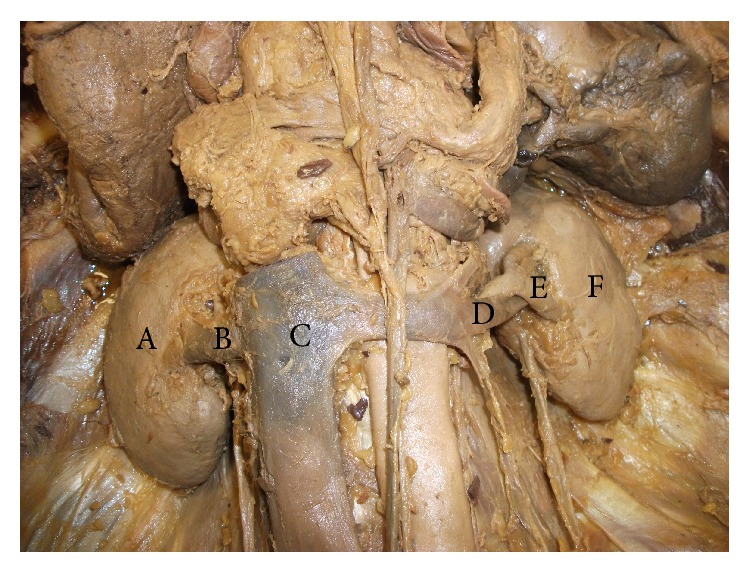
Right and left renal hila (anterior view). (A) Right kidney, (B) right renal vein, (C) inferior vena cava, (D) left renal vein, (E) left anterior inferior segmental renal artery, and (F) left kidney.

Thus, in this cadaveric case report, we have described a rare anatomical variant of a nutcracker phenomenon showing entrapment and compression of left renal vein between two renal segmental arteries at the hilum of left kidney. And, therefore, we have named this entity as hilar nutcracker phenomenon. The right renal hilum showed normal anatomical structures with renal vein, followed by renal artery and then pelvis of the ureter lying in anterior to posterior plane without any structural or course variations. The right and left kidneys were also visually normal looking with no apparent change in size and shape as well as no visible deformities in gross anatomical morphology (Figure 3). There were also no visible signs of varicocele or varicose veins on the lower extremities of the cadaver. Therefore, we felt that since there were no apparent signs of renal congestion in this cadaveric body, we should be addressing this particular anatomical anomaly showing compression of left renal vein between two segmental renal arteries as a nutcracker phenomenon rather than nutcracker syndrome in this cadaveric case report.

## 5. Discussion

The nutcracker phenomenon or nutcracker anatomy as an abnormal structural variation not always gives rise to clinical syndrome. And these anatomic abnormalities showing nutcracker phenomenon can be seen in normal individual with absolutely no clinical manifestations. Therefore, the patients with distinctive clinical symptoms along with noticeable anatomical variation displaying a nutcracker arrangement are the only patients diagnosed with nutcracker syndrome [3, 25, 26]. In this case report, we have presented an unusual case of nutcracker phenomenon which showed left renal vein getting entrapped between the anterior inferior and posterior segmental renal arteries. And along this course, the left anterior inferior segmental renal artery compresses the left renal vein due to its serpentine path at the hilum of left kidney. So, in order to distinguish such a rare variant of nutcracker phenomenon from usual anterior (classic) or posterior (retroaortic) nutcracker phenomenon, it should be called as hilar nutcracker phenomenon based on the anatomical location of the anomaly. Thus, reporting such a complex and rare anatomical variant of renal hilar vessels demonstrating nutcracker phenomenon is very significant from clinical perspective in terms of medical diagnosis and surgical approach.

The entrapment of renal vein in the two arms of the fork leads to nutcracker phenomenon. However, only few patients manifest the clinical sign and symptoms of renal vein compression. Therefore, the nutcracker syndrome and its related pathophysiology are difficult to understand or comprehend and so they remain elusive [3, 18]. Out of several theories presented in the medical literature, one of the theories states that posterior nephroptosis or renal ptosis can result in venous hypertension due to left renal vein getting stretched over the aorta causing its compression [13, 15, 27]. It is also proposed that, in the nutcracker syndrome, the abnormal branching of the superior mesenteric artery may cause an elevation in the pressure gradients between the proximal segment of the left renal vein and inferior vena cava [13]. Furthermore, in normal physiological condition, the pressure difference of less than 1 mmHg exists between the left renal vein and the inferior vena cava and in the nutcracker syndrome this pressure difference is more than 3 mmHg. This increase in pressure difference between the left renal vein and inferior vena cava promotes venous congestion leading to abnormal development of collateral branches between the renal sinus and the renal calyces causing hemorrhage [8]. One study also suggested that, in nutcracker syndrome patients, venous hypertension may cause hematuria due to disruption of the thin walled septum that separates the veins and the collecting ducts in the renal fornix. Also, the pooling of blood in the gonadal veins may lead to pelvic congestion in patients with nutcracker syndrome [8, 13].

In normal anatomical situation the superior mesenteric artery forms almost a 90° angle with the abdominal aorta and this structural organization protects the left renal vein from getting compressed by the superior mesenteric artery. However, in anterior nutcracker syndrome, the above angle is less than 90° and this reduction of angle or space between superior mesenteric artery and abdominal aorta may result in compression of left renal vein. The superior mesenteric artery then follows a very steep path downward resulting in entrapment of the left renal vein in between two handles of the nutcracker tool formed by abdominal aorta and superior mesenteric artery. Such abnormality in the anatomical arrangement often leads to the compression of the left renal vein between abdominal aorta and superior mesenteric artery. Thus, this above illustration gives a classical description of anterior nutcracker syndrome [1]. Moreover, in the sagittal plane, the angle between the abdominal aorta and superior mesenteric artery measured is normally greater than 45°. And in nutcracker anatomy this angle is less than 35°. It is also proposed that in nutcracker syndrome the caliber of the lumen of the left renal vein is reduced by more than 50% from its normal diameter of 4-5 mm, while it crosses abdominal aorta [8].

In conclusion, the nutcracker phenomenon or syndrome denotes an anatomical abnormality showing left renal vein getting trapped and compressed from surrounding structure. It results in renal hilum dilatation, increased left renal vein pressure, and congestion in the left kidney. The nutcracker phenomenon could be asymptomatic or could give rise to nutcracker syndrome with clinical manifestation such as hematuria, proteinuria, flank or abdominal discomfort or pain, varicocele in men, and pelvic congestion in women. In this report a rare case of hilar nutcracker phenomenon is presented. In hilar nutcracker phenomenon, the left renal vein is entrapped and compressed in between the anterior inferior and posterior segmental renal arteries at renal hilum. Also, the compression of left renal vein displaying a nutcracker phenomenon was due to unusual serpentine course of left anterior inferior segmental renal artery at the hilum of left kidney. Thus, the objective behind this report is to impart and share knowledge of anatomical variations in renal vasculature such as nutcracker phenomenon that not only will help in understanding pathophysiology behind renal disorders, but will also assist clinicians in diagnosing and treating such rare renal syndromes. Furthermore, highlighting such rare and critical anatomical variation in renal vasculature has academic as well as clinical significance due to increase in number of renal surgical, radiological, and transplant procedures being performed in recent years.
